# Health factors, stigma, and psychosocial resources associated with self-rated health: a cross-sectional analysis of the People Living with HIV Stigma Index 2.0 in Bolivia

**DOI:** 10.1016/j.lana.2026.101523

**Published:** 2026-06-04

**Authors:** Jason M. Lo Hog Tian, Mary Anne E. Roach, Carrie Lyons, Katherine Rucinski, Amrita Rao, John Mark Wiginton, Sarah M. Murray, Kaitlyn Atkins, Gnilane Turpin, Virginia Hilaquita Soto, Julio César Aguilera Hurtado, José Willan Montaño Ferrel, Martin Negrete, Keren Dunaway, Stefan D. Baral

**Affiliations:** a Department of Epidemiology, Bloomberg School of Public Health, Johns Hopkins University, Baltimore, USA; b Department of International Health, Bloomberg School of Public Health, Johns Hopkins University, Baltimore, USA; c Department of Mental Health, Bloomberg School of Public Health, Johns Hopkins University, Baltimore, USA; d Bolivian Network of People Living with HIV (REDBOL), Bolivia; e Universidad Autónoma Gabriel René Moreno, Santa Cruz de la Sierra, Bolivia; f Fundación Vencer, Asunción, Paraguay; g International Community of Women Living with HIV (ICW), London, UK

**Keywords:** HIV, Self-rated health, Quality of life, Comorbidities, Stigma, Latin America

## Abstract

**Background:**

As HIV has become an increasingly manageable health condition, improving quality of life for people living with HIV has been recognized as central to an effective HIV response. Self-rated health is a widely used, validated indicator of overall health and quality of life, yet factors shaping self-rated health among people living with HIV in Bolivia and across Latin America remain understudied.

**Methods:**

We analyzed data (n = 900) from the People Living with HIV Stigma Index in Bolivia conducted between June and December 2022. Multivariate logistic regression was used to assess how sociodemographic characteristics, health factors, stigma, and psychosocial resources were associated with self-rated health.

**Findings:**

Participants were an average of 35 years of age and identified as cisgender men (61%, n = 546), cisgender women (34%, n = 303), or transgender/non-binary (6%, n = 51). Fewer than half of participants rated their health as good (n = 384, 43%). In multivariate models, higher educational attainment (OR: 1.71; 95% CI: 1.10, 2.65), being virally suppressed (OR: 1.78; 95% CI: 1.14, 2.78), and greater HIV resilience (OR: 1.91; 95% CI: 1.39, 2.64) were significantly associated with increased odds of good self-rated health. Conversely, having comorbid health conditions (OR: 0.57; 95% CI: 0.43, 0.77) and greater internalized stigma (OR: 0.83; 95% CI: 0.76, 0.90) were associated with lower odds of good self-rated health.

**Interpretation:**

In addition to ensuring viral suppression, HIV programs and clinics must prioritize screening for and addressing comorbidities including cooccurring infections, mental health conditions, and noncommunicable diseases within environments free of stigma. Addressing internalized stigma and strengthening HIV-specific resilience may be critical for improving quality of life and supporting sustained engagement in HIV treatment and long-term well-being.

**Funding:**

This study was funded by the National Institute of Mental Health including R01MH140784 and the Center for HIV and Mental Health Stigma Elimination Strategies (CHIMES, P30MH136919), the National Institute of Allergy and Infectious Diseases (R01AI170249), the Global Fund country grant and regional project of the Alianza en Positivo (ALEP), the Bolivian Ministry of Health, and UNAIDS. KR was supported by the National Institute of Mental Health (K01MH129226). The funders had no role in the study design; collection, analysis, or interpretation of data; writing of the report; or in the decision to submit the paper for publication.

**Research in context**

**Evidence before this study**

With the success of antiretroviral therapy, HIV has become a manageable health condition with people with HIV living lifespans comparable to those without HIV. However, there is an increasing need to look beyond HIV treatment outcomes and understand broader indicators of health-related quality of life to ensure people living with HIV can thrive. Self-rated health is a long-established indicator used in population health research to assess subjective overall health and wellbeing, yet the factors shaping self-rated health remain underexplored among people living with HIV, particularly across low- and middle-income countries.

A search of PubMed was conducted from inception to November 2025 without language restrictions to identify studies that examined any determinants or factors associated with self-rated health in Bolivia or the broader Latin America region (n = 9). Literature revealed that no studies had described correlates of self-rated health in Bolivia and the limited work on self-rated health across Latin America had been conducted in Brazil. Moreover, people living with HIV reported worse self-rated health than people without HIV, with the rate of good or better self-rated health ranging from 65% to 81% for people living with HIV across those studies. Socioeconomic factors including education level and socioeconomic status, psychosocial factors such as drug use and depression, and HIV-related factors such as viral load, AIDS-related symptoms, and treatment related side effects were associated with worse self-rated health. A baseline understanding of self rated health among people living with HIV in Bolivia is needed for characterizing locally relevant determinants of health and wellbeing and making robust, data-driven recommendations for intervention development.

**Added value of this study**

To our knowledge, this is the first study to examine correlates of self-rated health among people living with HIV in Bolivia. Self-rated health offers critical insights into the lived experience of people with HIV that are not captured by clinical HIV outcomes alone. Using data from nine Ministry of Health HIV surveillance centers across the country, this work provides insights on a range of health and social factors that have not been previously studied in this context. The value added by this study lies in introducing a quality-of-life lens to HIV research and policy and program discussions in Bolivia, generating country-specific evidence that can guide stigma reduction strategies, psychosocial support initiatives, and integrated care approaches. It lays foundational groundwork for a research and program agenda focused on person-centered HIV care in a region where such data remain scarce. It also provides specific targets for the development and implementation of actionable and contextually relevant solutions to improving health-related quality of life for people living with HIV.

**Implications of all the available evidence**

With the majority of HIV programming and services focused on diagnosis and prevention, people already living with HIV risk being left behind, especially in regions of the world with less resources or investment in HIV research and programming. This is evidenced by a dearth of available data on health-related quality of life indicators such as self-rated health in people living with HIV in countries across Latin America. Existing evidence suggests that there are key determinants across socioecological levels which may serve as modifiable targets for intervention to improve self-rated health. This study provides supporting evidence for some of these associated factors while highlighting new health and social factors that may also play a key role in a person's self-rated health. Taken together, this study and the existing body of evidence underscore that improving health-related quality of life for people living with HIV requires more than a focus on HIV outcomes. As Bolivia continues to expand HIV care and treatment, incorporating screening and care for other comorbid conditions, creating an environment free of stigma, and building individual- and community-level resilience can support a more person-centered HIV response focused not only on survival, but on enabling people to thrive while living with HIV.

**Search strategy**

(“Human immunodeficiency virus” OR HIV) AND (“Self-rated health”) AND (Argentina OR Bolivia OR Brazil OR Chile OR Colombia OR “Costa Rica” OR Cuba OR “Dominican Republic” OR Ecuador OR “El Salvador” OR Guatemala OR Honduras OR Mexico OR Nicaragua OR Panama OR Paraguay OR Peru OR “Puerto Rico” OR Uruguay OR Venezuela OR “Latin America” OR “South America”)

## Introduction

As HIV has become an increasingly manageable chronic condition in many settings, there is growing recognition that health systems should look beyond HIV treatment adherence and viral suppression to address broader indicators of wellbeing for people living with HIV.^1^ Despite biomedical advances, people living with HIV still report lower health-related quality of life compared to other reproductive-aged adults, even among people living with HIV who are virally suppressed.^2^ Self-rated health, sometimes known as overall health or perceived health, is measured using one question asking participants to rate their general health from “poor” to “excellent” and has been used extensively both as a proxy for more objective health and as a measure of health-related quality of life.^3^ This one question has demonstrated robust ability to predict key health outcomes including mortality, morbidity, and objective health status.^3^ Self-rated health has the advantage of being low-burden, cross-culturally interpretable, and highly sensitive to changes in both health status and psychosocial context.^4^ Evidence has shown that higher self-rated health is linked to HIV care engagement, HIV treatment adherence, viral load suppression, lower rates of comorbidity, and better mental health.^5^, ^6^, ^7^, ^8^ Thus, seeking to improve self-rated health may not only improve general wellbeing and functioning but also serve as a mechanism for advancing HIV treatment targets.

In Latin America, much of the HIV research and programming has focused on countries with high HIV prevalence such as Brazil, Chile, or Peru.^9^ The lived experiences of people living with HIV in Bolivia where social, economic, and political context may shape health in unique ways remain understudied. HIV prevalence in Bolivia remains relatively low compared to other countries in South America at 0.3%, with 31,000 people estimated to be living with HIV.^10^ Despite antiretroviral therapy (ART) being made available for free to all citizens as mandated by law, just over half of the people living with HIV in Bolivia are on ART and 83% of people on ART have an undetectable viral load.^10^ These gaps in treatment coverage, alongside persistent stigma and socioeconomic inequalities, may influence how people living with HIV experience and evaluate their health.

Evidence on self-rated health among people living with HIV in Latin America remains limited. Only two studies from Brazil found socioeconomic status, sexual activity, HIV symptoms, substance use, and depression were associated with self-rated health in people living with HIV.^8^^,^^11^ However, these studies did not examine a broader range of health and social determinants that may shape perceptions of health among people living with HIV, including HIV stigma, psychosocial resources, and the burden of co-occurring health conditions. Understanding how these factors jointly influence self-rated health is particularly important in settings such as Bolivia where structural barriers, stigma, and uneven access to care may affect both clinical outcomes and perceived wellbeing.

**Fig. 1:**
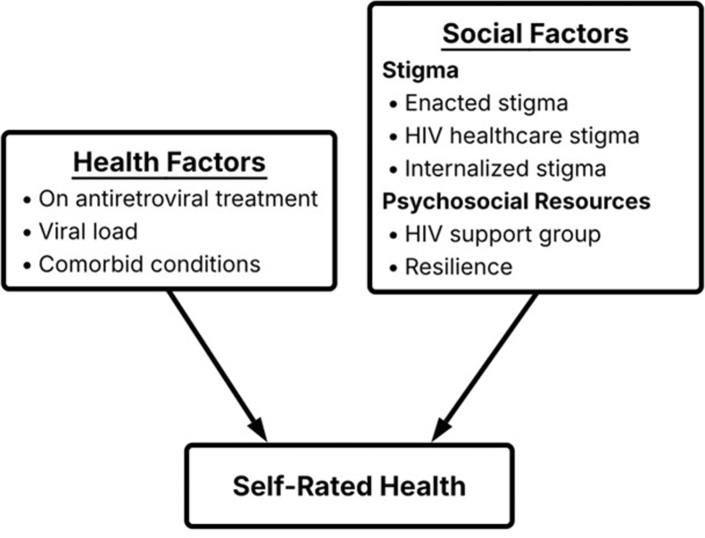
Conceptual framework of health and social factors affecting self-rated health (based on Garbarski, 2016).

In a review of extant literature around self-rated health, Garbarski (4) constructed a conceptual framework that highlighted the most commonly studied factors associated with self-rated health, with health and social factors emerging as two major dimensions. The health dimension predominantly consists of the health conditions, diseases, symptoms, functional status, and health service use that people consider when forming an assessment of their health. The social dimension includes social factors associated with health as well as differences in health evaluation across social groups. Fig. 1 depicts an adapted version of this conceptual framework, incorporating health and social factors specific to the lived experiences of people with HIV. Under health factors, people may consider HIV-specific clinical indicators such as medication adherence and viral load in their assessment of health. Furthermore, as people are aging with HIV, the cumulative burden of comorbid health conditions such as non-communicable diseases and mental health challenges may increase rapidly which can also contribute to overall health.^9^^,^^12^ Research has demonstrated how both clinical HIV indicators and comorbid conditions have strong links with self-rated health.^6^ Under social factors, HIV-related stigma remains a central barrier to good health and wellbeing for people living with HIV, and experiences with different types of HIV-related stigma have been associated with worse self-rated health.^13^^,^^14^ Conversely, research has demonstrated how psychosocial resources such as HIV-related resilience or social support can reduce the impact of negative risk factors and improve overall health.^15^^,^^16^

Characterizing how both health and social factors shape self-rated health among people living with HIV can inform more holistic, equity-oriented interventions both in Bolivia and in the broader Latin American context, especially in areas where ART coverage and health system reach remain uneven. In response, this study aimed to examine how health factors including clinical HIV indicators and comorbid health conditions, and social factors including HIV-related stigma and psychosocial resources are associated with self-rated health for people living with HIV in Bolivia.

## Methods

### Study design

This study uses cross-sectional data (n = 945) from the People Living with HIV Stigma Index 2.0 in Bolivia. The original People Living with HIV Stigma Index was developed in 2008 by the Global Network of PLHIV (GNP+), the International Community of Women Living with HIV (ICW), International Planned Parenthood Foundation, and the Joint United Nations Programme for HIV and AIDS (UNAIDS) and aimed to measure stigma and discrimination from the perspective of people living with HIV.^17^ In 2018, an updated version of the tool was launched that reflected shifts in the HIV epidemic and the global response.^18^ The HIV Stigma Index methodology is grounded in the principles of greater involvement and meaningful engagement of people living with HIV (GIPA/MIPA), with people living with HIV actively engaged throughout the research process including survey adaptation, participant recruitment, data collection through peer interviewers, and interpretation of results.^18^, ^19^, ^20^

The study was conducted across the nine departments of Bolivia between June and December 2022. Participants were recruited primarily through Departmental Surveillance, Information and Referral Centres (Centros Departamentales de Vigilancia, Información y Referencia; CDVIR), which are specialized HIV services within the Bolivian Ministry of Health responsible for HIV testing, treatment, and follow-up care. One CDVIR located in each departmental capital participated in the study (La Paz, Cochabamba, Santa Cruz, Chuquisaca, Oruro, Potosí, Tarija, Beni, and Pando). Recruitment was complemented by outreach through civil society organizations serving people living with HIV and key populations. The sample distribution across departments was guided by national HIV surveillance data to broadly reflect the geographic distribution of HIV cases in Bolivia.

Participants were eligible for the study if they were 18 years or older, living with HIV, and aware of their HIV status for at least one year. Recruitment employed two complementary approaches. First, place-based sampling was conducted at CDVIR clinics using systematic probability sampling (approximately 65% of the total sample). Recruitment intervals were determined based on the estimated daily number of eligible patients attending each clinic, and trained interviewers approached individuals at regular intervals (e.g., every second or third eligible patient) during recruitment periods. Second, limited chain-referral sampling was used to recruit individuals from key populations disproportionately affected by HIV, including men who have sex with men, transgender women, sex workers, and people who use drugs (approximately 35% of the total sample). Initial participants (“seeds”) were identified through community organizations and invited to refer other eligible individuals within their networks.

The survey was administered face-to-face by trained peer researchers who were themselves people living with HIV and affiliated with community organizations. Interviews were conducted in private spaces and lasted approximately 2 hours. Participants were compensated 50 Bolivianos for participating. The survey was hosted on the RED-Cap (Research Electronic Data Capture) online survey platform. Survey data for each participant was coded using a unique study ID and anonymized. Data was stored securely and confidentially on an encrypted server at Johns Hopkins University and will only be accessible by authorized personnel. Informed written consent was obtained from all participants. This study was conducted in collaboration between Johns Hopkins University, Universidad Autónoma Gabriel René Moreno (UAGRM), Fundación Hábitat Verde (FHV), and the Red Nacional de Personas con VIH de Bolivia (REDBOL). REDBOL is the derivative holder of the copyright of the written work entitled “INDX 2.0 Bolivia” registered before the Servicio Nacional de Propiedad Intelectual (SENAPI) through Administrative Resolution No. 1-780/2023 dated April 4, 2023 (Procedure No. DA-S 200522/2022). Institutional review board approval was obtained from Universidad Autónoma Gabriel René Moreno (#127/2021).

### Measures

All variables in this study were self-reported. Sociodemographic characteristics were included as covariates. The primary exposures were health factors (continuous HIV treatment, viral load, and comorbid health conditions), HIV stigma (enacted stigma, HIV healthcare stigma, and internalized stigma), and psychosocial resources (HIV resilience and being a member of an HIV support group). The outcome was self-rated health (good versus poor/fair).

#### Sociodemographic characteristics

Sociodemographic characteristics were collected including age, gender, education (less than high school, completed high school, education beyond high school), employment (full-time, part-time, self-employed, informal part-time, not employed/retired), and ability to meet basic needs such as food or shelter in the past 12 months (basic needs met, unmet some of the time, unmet most of the time). For analyses, participants who identified as gender minorities were combined due to small sample sizes.

#### Health factors

To determine those who were continuously on HIV treatment, participants were asked if they had ever been on HIV treatment (yes/no) and if they had ever interrupted or stopped HIV treatment (yes, no, don't know). If a person responded “yes” to having ever been on HIV treatment and “no” to having ever interrupted or stopped treatment, they were classified as continuously on treatment with no interruptions. Participants who had never been on HIV treatment, ever interrupted treatment, or did not know were classified as not continuously on treatment.

The viral load variable was based on asking participants if they had an undetectable viral load test in the last 12 months. Participants could answer “yes”, “no, the virus was detectable”, “no, I have not had a test in the last 12 months”, “no, I had a test and am waiting for the results”, “no, I have never had a viral load test”, and “I don't know what viral load or viral suppression are”. Participants who did not have a test, were waiting for test results, or did not know were combined as “don't know”.

To assess comorbid health conditions, participants were asked if they had been diagnosed with any of the following conditions in the past 12 months including tuberculosis, viral hepatitis, sexually transmitted infections (e.g., herpes, gonorrhea, chlamydia, syphilis), mental health conditions (e.g., anxiety, depression, insomnia, post-traumatic stress disorder), noncommunicable diseases, or alcohol/drug dependency syndrome(s). Comorbid health conditions were categorized into diagnosed with no conditions versus having been diagnosed with one or more conditions.

#### HIV stigma

Enacted HIV stigma was assessed using 11 questions from the People Living with HIV Stigma Index 2.0.^18^ Participants were asked about stigma or discrimination experienced because of their HIV status and were able to answer with “yes, within the last 12 months”, “yes, but not within the last 12 months”, and “no”. The items were dichotomized by combining the two “yes” options (ever experienced). Participants who answered “yes” to at least one question were classified as having enacted stigma.

HIV-related healthcare stigma was assessed using seven questions from the People Living with HIV Stigma Index 2.0.^18^ Participants were asked “In the past 12 months, when seeking HIV specific health care, have you experienced any of the following from health facility staff working in the place you receive your HIV care” and were shown a list of seven healthcare stigma experiences with the option of responding with “yes” or “no”. Participants who answered “yes” to at least one question were classified as having HIV healthcare stigma.

Internalized stigma was measured using the Internalized AIDS-Related Stigma Scale (IA-RSS).^21^ The scale contained 6 items such as “It is difficult to tell people about my HIV diagnosis” or “I feel guilty that I am HIV positive”. Participants can respond with “agree” or “disagree” and a total score was calculated by summing the “agree” responses. Internal consistency for the IA-RSS was adequate with a Cronbach's alpha of 0.75.

#### Psychosocial resources

To measure the availability of HIV-specific social support or support networks, participants were asked if they were a member of a network or support group of people living with HIV and were able to answer yes/no.

Resilience was measured using the People Living with HIV Resilience Scale.^22^ Participants were shown 10 items such as “my self-confidence” or “my ability to cope with stress” and asked if these items were positively, negatively, or not affected by their HIV status in the past 12 months. Answering positively was counted as plus one point, negatively as minus one point, and not affected as zero points. A total score was calculated by summing the responses to all questions, and the total score dichotomized into “positively affected” (score >0) versus “negatively or not affected” (score ≤0). Internal consistency was good with a Cronbach's alpha of 0.83.

#### Self-rated health

To measure self-rated health, participants were asked “In general, how would you describe your health at the moment” and could respond with “good”, “fair”, or “poor”.^3^ For analysis, self-rated health was dichotomized as “good” versus “poor/fair”.

### Statistical analyses

Participants who did not have complete data for the variables in these analyses were excluded, leaving final sample of n = 900 (people who preferred not to answer: 10 for gender, 10 for HIV healthcare stigma, and 29 for viral load; participants may have preferred not to answer multiple questions). Descriptive statistics (proportions, means, standard deviations) were used to summarize the demographic characteristics and variables of interest. Bivariate and multivariate logistic regression was used to identify variables independently associated with good self-rated health. Health and social factors were entered as exposures in the model with sociodemographics being entered as covariates and self-rated health as the outcome. Odds ratios, 95% confidence intervals, and p-values were reported. An association was deemed significant with a p-value less than 0.05. Hosmer–Lemeshow tests were used to assess model fit, with a p-value greater than 0.05 indicating a good fit. A sensitivity analysis was conducted by running all models using the full viral load variable to determine if the merging of response options led to significantly different results. All statistical analyses were conducted using IBM SPSS Statistics version 24.^23^

### Role of the funding

The funders had no role in the study design; collection, analysis, or interpretation of data; writing of the report; or in the decision to submit the paper for publication.

## Results

### Participant characteristics

Table 1 reports key demographic characteristics and other variables of interest among the participants included in this analysis (n = 900/945, 95%). Participants were recruited from all nine departmental capitals of Bolivia. The largest proportions of participants were from Santa Cruz (40%, n = 361), La Paz (24%, n = 217), and Cochabamba (23%, n = 204), while smaller proportions were recruited from Chuquisaca (4%, n = 33), Beni (3%, n = 27), Tarija (2%, n = 22), Potosí (2%, n = 20), Oruro (1%, n = 11), and Pando (1%, n = 5). Participants were an average of 35 years of age and mostly identified as cisgender men (61%, n = 546), had high school education or greater (79%, n = 715), were employed (74%, n = 490), and had unmet basic needs most of the time (49%, n = 445). Most participants were continuously on HIV treatment (59%, n = 532) and had an undetectable viral load (68%, n = 612). Almost half of the sample (46%, n = 416) reported at least one comorbid health condition. These comorbidities included mental health conditions (29%, n = 261), sexually transmitted infections (16%, n = 146), noncommunicable diseases (10%, n = 88), alcohol/drug dependency (9%, n = 82), tuberculosis (5%, n = 47), and viral hepatitis (2%, n = 17). Participants endorsed at least one item for enacted HIV stigma (57%, n = 515) and HIV-related healthcare stigma (40%, n = 358). The mean score for the internalized stigma scale was 3.42 (SD: 1.80, range: 1–6). Over one third (37%, n = 329) were members of an HIV support group and two thirds (65%, n = 586) had been positively impacted by their HIV status (HIV resilience). Lastly, 5% (n = 49) of people rated their health as poor, 52% (n = 467) as fair, and 43% (n = 384) as good.

**Table 1:** Demographic characteristics and variables of interest (n = 900).

	n or mean	% or SD
Sociodemographic characteristics		
Age	35.1	10.9
Gender		
Cisman	546	61%
Ciswoman	303	34%
Transgender/non-binary	51	6%
Education		
<High school	185	21%
High school	373	41%
>High school	342	38%
Employment		
Full-time	233	26%
Part-time	129	14%
Self-employed	138	15%
Informal part-time	175	19%
Not employed/retired	225	25%
Basic needs (past 12 months)		
Basic needs met	29	3%
Unmet some of the time	426	47%
Unmet most of the time	445	49%
Health factors		
Continuously on HIV treatment		
No	368	41%
Yes	532	59%
Undetectable viral load		
No	130	14%
Don't know	158	18%
Yes	612	68%
Comorbid health conditions		
0	484	54%
1+	416	46%
Stigma		
Enacted stigma		
No	385	43%
Yes	515	57%
HIV healthcare stigma		
No	542	60%
Yes	358	40%
Internalized stigma	3.4	1.8
Positive resources		
HIV support group		
No	571	63%
Yes	329	37%
Resilience (impact of HIV)		
Negatively or not affected	314	35%
Positively affected	586	65%
Outcome		
Self-Rated Health		
Poor	49	5%
Fair	467	52%
Good	384	43%

### Associations with self-rated health

Table 2 shows the results of the logistic regression showing associations of sociodemographic characteristics, clinical outcomes, stigma, and positive resources with self-rated health. Out of all the demographic characteristics, only education was significantly associated with self-rated health in the multivariate model, with participants with greater than high school education being more likely to have good self-rated health than those with less than high school education (OR: 1.71; 95% CI: 1.10, 2.65). Having an undetectable viral load was also associated with good self-rated health (OR: 1.78; 95% CI: 1.14, 2.78). People with one or more comorbid health conditions (OR: 0.57; 95% CI: 0.43, 0.77) and people with internalized stigma (OR: 0.83; 95% CI: 0.76, 0.90) were less likely to have good self-rated health. Lastly, those who were positively affected by their HIV status were more likely to have good self-rated health (OR: 1.91; 95% CI: 1.39, 2.64). We failed to reject the Hosmer–Lemeshow null hypothesis (Hosmer–Lemeshow chi-square = 4.03, p = 0.85) which indicates good model fit. Sensitivity analyses with the full viral load variable showed no significant differences in findings (see Supplementary Table S1).

**Table 2:** Bivariate and multivariate logistic regression with variables of interest predicting good self-rated health.

	Bivariate			Multivariate		
	OR	95% CI	p	OR	95% CI	p
Sociodemographic characteristics						
Age	1.01	(1.00, 1.02)	0.08	1.01	(0.99, 1.02)	0.42
Gender						
Cisman		ref			ref	
Ciswoman	0.78	(0.59, 1.04)	0.10	1.03	(0.73, 1.44)	0.88
Transgender/non-binary	0.86	(0.48, 1.54)	0.61	0.98	(0.52, 1.84)	0.95
Education						
<High school		ref			ref	
High school	1.33	(0.91, 1.93)	0.14	1.20	(0.79, 1.81)	0.40
>High school	2.43	(1.67, 3.54)	**<0.01**	1.71	(1.10, 2.65)	**0.02**
Employment						
Full-time		ref			ref	
Part-time	0.89	(0.58, 1.37)	0.59	0.98	(0.61, 1.57)	0.92
Self-employed	1.00	(0.66, 1.53)	1.00	1.05	(0.66, 1.67)	0.83
Casual/informal part-time	0.67	(0.45, 1.00)	**0.05**	0.74	(0.48, 1.16)	0.19
Unemployed/retired	0.80	(0.55, 1.16)	0.24	1.02	(0.67, 1.55)	0.93
Basic needs						
Basic needs met		ref			ref	
Unmet some of the time	1.11	(0.50, 2.45)	0.80	1.22	(0.51, 2.94)	0.65
Unmet most of the time	1.81	(0.82, 3.98)	0.14	1.55	(0.65, 3.73)	0.33
Health factors						
Continuously on HIV treatment						
No		ref			ref	
Yes	1.64	(1.25, 2.16)	**<0.01**	1.21	(0.90, 1.65)	0.21
Undetectable viral load						
No		ref			ref	
Don't know	1.08	(0.65, 1.79)	0.76	1.06	(0.62, 1.82)	0.83
Yes	2.17	(1.45, 3.26)	**<0.01**	1.78	(1.14, 2.78)	**0.01**
Comorbid health conditions						
0		ref			ref	
1+	0.50	(0.38, 0.65)	**<0.01**	0.57	(0.43, 0.77)	**<0.01**
Stigma						
Enacted stigma						
No		ref			ref	
Yes	1.64	(1.25, 2.16)	**<0.01**	0.92	(0.68, 1.24)	0.57
HIV healthcare stigma						
No		ref			ref	
Yes	0.73	(0.55, 0.95)	**0.02**	0.98	(0.71, 1.35)	0.89
Internalized stigma	0.75	(0.69, 0.81)	**<0.01**	0.83	(0.76, 0.90)	**<0.01**
Positive resources						
HIV support group						
No		ref			ref	
Yes	1.12	(0.85, 1.47)	0.43	0.90	(0.65, 1.24)	0.52
Resilience (impact of HIV)						
Negatively or not affected		ref			ref	
Positively affected	2.34	(1.75, 3.14)	**<0.01**	1.91	(1.39, 2.64)	**<0.01**

Bold values indicate p < 0.05.

## Discussion

The present study aimed to assess associations between health factors, HIV-related stigmas, and psychosocial resources on self-rated health in a large sample of people living with HIV in Bolivia. Overall, fewer than half of the participants reported good self-rated health, highlighting a substantial gap in perceived wellbeing among people living with HIV. This proportion appears lower than estimates reported in studies of people living with HIV in other Latin American settings, such as Brazil, where between approximately 65% and 81% of participants have reported good or better self-rated health.^8^^,^^11^ Differences in self-rated health across settings may reflect broader social and structural conditions including persistent socioeconomic inequalities, ongoing HIV-related stigma, and gaps in the HIV care continuum in Bolivia. In multivariable analyses, educational attainment, viral load status, comorbid health conditions, internalized stigma, and HIV resilience were all significantly associated with self-rated health. These findings underscore the multidimensional nature of self-rated health, shaped not only by clinical HIV indicators but also by other health and social factors that either hinder or promote wellbeing.

HIV treatment outcomes remain suboptimal in Bolivia, even with universal access to HIV medicines nationwide.^10^ Gaps along the HIV care continuum, including challenges with sustained treatment engagement and viral suppression, may contribute to poorer self-rated health among people living with HIV. Consistent with this, participants who reported having an undetectable viral load had significantly higher odds of good self-rated health. This finding aligns with previous research showing that successful HIV treatment and viral suppression are associated with improved health-related quality of life and wellbeing.^6^ On the other hand, studies have also found how health-related quality of life can reciprocally impact outcomes such as healthcare engagement, adherence, and long-term health management that are important for HIV epidemic control and the care continuum.^24^ However, good HIV outcomes alone do not equate to good quality of life and characterizing the other health and social factors that most greatly affect self-rated health provides potential targets for future interventions to improve both the quality and ultimately quantity of life for people living with HIV.

Out of all the sociodemographic characteristics, higher educational attainment was associated with better self-rated health. This aligns with existing research linking education to improved healthcare access, psychosocial resources, and health literacy.^25^ In the Bolivian context, educational disparities are closely tied to socioeconomic inequality, with Indigenous populations and rural communities facing higher barriers to formal education and health access.^26^^,^^27^ Reciprocally, people with better health outcomes and self-rated health may also be able to attend school for longer or be more likely to go on to higher education. Upstream investments in equitable educational access and lifelong learning opportunities, including vocational training and occupational pathways, may be an important part of broader strategies to improve health and quality of life for people living with HIV.

Comorbid health conditions were significantly associated with poorer self-rated health, consistent with evidence that has linked comorbidities with HIV risk, medication adherence, stigma, and quality of life.^28^^,^^29^ As people have access to HIV treatment and can live longer lives, other health conditions including non-communicable diseases, other viral and bacterial infections, and mental health challenges are increasingly affecting the daily lives of people living with HIV.^9^^,^^12^ Interventions focused on effectively supporting people living with HIV should account for these overlapping challenges, particularly as the people living with HIV continue to age and face increasing burden of non-communicable diseases.

Internalized stigma was the only form of stigma significantly associated with self-rated health in this study. This finding coincides with previous literature linking internalized stigma to a range of HIV outcomes, mental health, and self-rated health.^13^^,^^30^ People can experience internal shame and guilt associated with having HIV which may affect self-rated health by lowering health expectations (e.g., “I deserve to be sick”) or by facilitating negative social comparisons.^31^^,^^32^ This is especially problematic since levels of HIV stigma in Bolivia remains high and continues to be one of the most salient issues with which people living with HIV struggle.^33^, ^34^, ^35^ A systematic review of internalized stigma interventions highlights how interventions targeting a mixture of structural-level factors such as ART provision and social empowerment, and individual-level factors such as resilience were most effective.^36^ Integrated HIV care models that actively address internalized stigma as part of routine care through resilience-building, peer support, and structural interventions are needed to challenge the social conditions producing shame and guilt around living with HIV.

HIV-related resilience, measured as being positively affected by one's diagnosis, was significantly associated with better self-rated health. This supports growing evidence showing that resilience—not just in general, but specific to living with HIV—is a critical positive resource.^16^^,^^22^ Resilient individuals may be better able to reframe health challenges and assess their health in light of personal growth and coping.^4^ This study adds to evidence that self-rated health is shaped not only by illness and adversity, but also by internal strengths. While much research has focused on stigma and risk, there is a need for greater investment in understanding and fostering resilience among people living with HIV in Bolivia and across Latin America.

These findings should be interpreted considering several limitations. First, the study's cross-sectional design means we cannot draw causal inferences about the directionality of associations between these clinical and social factors and self-rated health. It is possible, for example, that individuals with poorer self-rated health may also be more likely to report higher levels of internalized stigma or lower resilience. The study sample was recruited through convenience sampling at HIV surveillance centers which may limit the representativeness of the findings. Individuals who are engaged in care or connected to these centers may differ systematically from people living with HIV who are not regularly accessing HIV services. Furthermore, although participants were recruited from all nine departmental capitals, the sample was concentrated in the central axis departments of La Paz, Cochabamba, and Santa Cruz, where HIV services and population density are greater. As a result, the findings may not fully reflect the experiences of people living with HIV in smaller or more resource-constrained departments. As data were collected through face-to-face self-report interviews, responses may be subject to information bias, including social desirability bias when reporting sensitive experiences such as stigma, as well as potential recall bias for past events. However, interviews were conducted by trained peer interviewers living with HIV, which may have facilitated trust and more open disclosure of experiences among participants. Although the self-rated health measure is widely validated and predictive of outcomes including mortality, morbidity, and objective health status,^3^ it is a single-item measure and may not capture nuances across different health domains. Further work to parse out how these health and social factors affect different aspects of physical and mental health is needed to further inform interventions and policies aiming to address specific domains of quality of life. There are also other important psychological, social, and structural variables that were not examined here that may have a significant impact in shaping a person's self-rated health. For example, factors such as availability of basic needs, social support, and healthcare empowerment may shape how people living with HIV rate their health.^15^^,^^37^^,^^38^ The absence of these variables in the present analysis may result in residual confounding or an incomplete understanding of the broader determinants influencing self-rated health.

Overall, this work identifies modifiable factors that require further investigation as potential key targets for intervention or policy changes aimed at improving the lives of people living with HIV in Bolivia. In clinical settings, integrating routine screening for comorbid health conditions including mental health disorders, noncommunicable diseases, and co-occurring infections within HIV care could help identify individuals at higher risk of poor self-rated health and facilitate more comprehensive management. HIV services should also prioritize stigma-free environments, with special attention on internalized stigma, through provider training, patient-centered care practices, and mechanisms to identify and address stigma within healthcare settings. In addition, resilience-building approaches such as peer support programs, strengths-based counseling, and community-led support groups may help individuals develop coping strategies and improve perceptions of health and wellbeing.^22^^,^^39^ Together, these approaches could support a more person-centered HIV care model that moves beyond viral suppression alone to address the broader determinants of quality of life. These findings also highlight the potential value of incorporating simple patient-reported measures such as self-rated health into routine HIV program monitoring. As a brief and validated indicator of overall wellbeing, self-rated health could help programs identify unmet needs that may not be captured by biomedical indicators alone. By examining self-rated health alongside traditional HIV care indicators, this study offers new insights into the broader experiences and unmet needs of people living with HIV in Bolivia and Latin America more broadly and highlights opportunities for more holistic, person-centered care strategies.

### Conclusion

This study highlights the health and social factors that shape how people living with HIV in Bolivia perceive their health. Strengthening integrated care models that address mental health, co-occurring conditions, and stigma and incorporating resilience-building and peer support strategies into routine care are essential next steps for improving self-rated health. As Bolivia and countries across Latin America continue to expand HIV treatment and care, the results presented here suggest that health-related quality of life should be recognized as both a means and an end that is critical for achieving sustained engagement in treatment and ensuring that people living with HIV are not only surviving, but thriving.

## Contributors

JML: Conceptualization, Formal analysis, Writing—original draft; SDB: Conceptualization, Methodology, Funding acquisition, Project administration, Supervision, Writing—review & editing; VHS, JCAH, MN, YC: Investigation, Project administration, Data curation, Writing—review & editing; MAE, CL, KR, AR, JMW, SMM, KA, GT, KD: Writing—review & editing. JML and MAE had full access to the underlying data and verified the data. All authors had full access to all the data in the study and had final responsibility for the decision to submit for publication.

## Data sharing statement

The datasets used and/or analysed during the current study are available from the corresponding author on reasonable request.

## Declaration of interests

The authors declare that they have no competing interests.
