# Supplementary material for: Health factors, stigma, and psychosocial resources associated with self-rated health: a cross-sectional analysis of the People Living with HIV Stigma Index 2.0 in Bolivia
Source: Lancet Reg Health Am. 2026 Jun 4;61:101523. doi: 10.1016/j.lana.2026.101523 (PMC13266133; doi:10.1016/j.lana.2026.101523)
Supplement: Supplementary Table S1 [file mmc1.pdf]

Supplementary Table 1 Sensitivity analysis of multivariable logistic regression with full viral load variable

	OR	95% CI	*p*
**Sociodemographic Characteristics**			
Age	1.01	(0.99, 1.02)	0.46
Gender			
Cisman		ref	
Ciswoman	1.03	(0.74, 1.45)	0.85
Transgender/non-binary	0.95	(0.50, 1.79)	0.87
Education			
< High school		ref	
High school	1.21	(0.80, 1.83)	0.37
> High school	1.76	(1.13, 2.75)	**0.01**
Employment			
Full-time		ref	
Part-time	0.98	(0.61, 1.57)	0.92
Self-employed	1.05	(0.66, 1.67)	0.84
Casual/informal part-time	0.75	(0.48, 1.17)	0.20
Unemployed/retired	1.04	(0.68, 1.58)	0.87
Basic needs			
Basic needs met		ref	
Unmet some of the time	1.20	(0.50, 2.87)	0.69
Unmet most of the time	1.51	(0.63, 3.64)	0.36
**Clinical Outcomes**			
Continuously on HIV treatment			
No		ref	
Yes	1.22	(0.90, 1.66)	0.20
Undetectable viral load			
No		ref	
Yes	1.78	(1.14, 2.78)	**0.01**
Waiting for results	1.36	(0.72, 2.59)	0.35
Haven't had a test in last 12 months	1.02	(0.49, 2.12)	0.97
Don't know what viral load or viral suppression are	0.54	(0.19, 1.55)	0.25
Comorbid health conditions			
0		ref	
1+	0.57	(0.42, 0.77)	**<0.01**
**Stigma**			
Enacted stigma			
No		ref	
Yes	0.91	(0.67, 1.23)	0.53
HIV healthcare stigma			
No		ref	
Yes	0.96	(0.70, 1.32)	0.81
Internalized stigma	0.82	(0.75, 0.90)	**<0.01**
**Positive Resources**			
HIV support group			
No		ref	
Yes	0.89	(0.64, 1.23)	0.48
Resilience (impact of HIV)			
Negatively or not affected		ref	
Positively affected	1.89	(1.37, 2.61)	**<0.01**
